# TNBC Spatial Transcriptomic Analysis across Clinical States Reveals Subtype-Specific Networks and Immunosuppressive Niches

**DOI:** 10.1158/2767-9764.CRC-25-0808

**Published:** 2026-05-29

**Authors:** Fengyuan Huang, Nitish Kunte, Himani Khurana, Clayton C. Yates, Deepa Bedi

**Affiliations:** 1 Center for Biomedical Research, Tuskegee University, Tuskegee, Alabama.; 2 Department of Pathology and Oncology, John Hopkins University, Baltimore, Maryland.

## Abstract

Breast cancer is a heterogeneous disease composed of distinct molecular subtypes that influence prognosis and treatment response, with subtype discordance between primary and metastatic tumors contributing to therapeutic failure. Using GeoMx spatial transcriptomics, we profiled nonmetastatic primary tumors, metastatic primary tumors, and lymph node (LN) metastases to characterize transcriptional and immune–spatial changes during disease progression. Comparative analysis revealed strong tumor microenvironment reprogramming across clinical states. In stromal regions of metastatic tumors, we observed enrichment of extracellular matrix remodeling, tumor-associated macrophage activity, and epithelial-to-mesenchymal transition–related pathways. In epithelial compartments, nonmetastatic and LN tumors showed a secretory–proliferative program, whereas primary metastatic tumors were enriched for antigen presentation and interferon (IFN) signaling. Immune deconvolution demonstrated distinct immune landscapes, with nonmetastatic tumors enriched for innate immune cells, primary metastatic tumors showing increased T- and B-cell infiltration, and LN metastases exhibiting an immunosuppressive microenvironment dominated by M2-like macrophages and memory B cells (*P* < 0.05). Subtype analysis revealed frequent triple-negative breast cancer (TNBC) subtype switching during LN metastasis, with 70% of cases transitioning from nonbasal subtypes to unspecified or immunomodulatory subtypes, both associated with poorer prognosis (HR = 1.8; 95% CI, 1.2–2.9). Spatial immune profiling further identified “excluded” and “ignored” tumors lacking T-cell infiltration, whereas “inflamed” tumors retained active cytotoxic immune responses. Integrating molecular subtype, immune infiltration, and spatial architecture, we developed a novel framework termed immuno-spatial molecular subtypes (ISMS), which captures TNBC heterogeneity and immune–spatial plasticity across disease progression.

**Significance:**

TNBC progression is poorly understood, particularly how tumor and immune ecosystems evolve. By applying spatial transcriptomics on tissues, we uncover that nonmetastatic tumors displayed metabolic programs and a more coordinated immune–proliferation balance. Metastatic primary showed stronger IFN and antigen-presentation programs, whereas LN metastases showed an immune-excluded niche. Our discovery that immune phenotypes diverge from canonical subtypes led to development of ISMS, a framework that captures TNBC plasticity more accurately than subtype alone.

## Introduction

Breast cancer is a highly heterogeneous disease classified into several molecular subtypes based on the expression of hormone receptors [estrogen receptor (ER) and progesterone receptor (PR)] and HER2/neu (1), as well as genomic and transcriptomic profiles. These subtypes include luminal A (ER+/PR+ and Ki67-low), luminal B (ER+/PR+, HER2±, and Ki67-high), HER2-enriched (HER2+), basal-like, claudin-low, and normal breast–like (2, 3). These classifications reflect not only the intrinsic biology of tumors but also their distinct tumor microenvironments (TME), contributing to differences in clinical behavior, therapeutic response, and prognosis.

Triple-negative breast cancer (TNBC), which lacks ER, PR, and HER2 expression, represents 10% to 20% of all breast cancers and is associated with an aggressive clinical trajectory and poor prognosis (2, 4). TNBC itself is a highly heterogeneous disease, and according to the Lehmann classification (5), it can be subdivided into distinct molecular subtypes based on transcriptomic profiling: basal-like 1 (BL1), basal-like 2 (BL2), mesenchymal (M), mesenchymal stem-like (MSL), immunomodulatory (IM), and luminal androgen receptor (LAR). These molecular subtypes are also associated with distinct heterogeneous TMEs and differential response to therapy (6, 7). Studies have shown that tumors evolve through continuous interaction with immune and nonimmune cells of the stroma within the TME, which drives plasticity, including subtype switching during progression and therapy complicating risk stratification and precision treatment (8, 9).

Lymph node (LN) involvement represents a pivotal step in TNBC progression and patient management. Yet the nonmetastatic TNBC phenotype, tumors with no clinical or pathologic evidence of nodal spread, seems to be uncommon and remains poorly defined at the molecular and spatial–immune levels. A key unmet need is to understand how the three clinical states, represented by (i) nonmetastatic, (ii) metastatic primary, and (iii) LN metastases, differ in transcriptional programs, immune composition, and spatial organization of antitumor versus immunosuppressive niches.

The advent of high-plex spatial transcriptomics has revolutionized the measurement of gene expression within intact tissue architecture, permitting simultaneous assessment of subtype identity, pathway activity, and immune positioning at scale (10–12). Coupling these data with computational deconvolution has enabled immune–spatial phenotyping, offering a route to actionable pathways/biomarkers that reflect where and how immune cells interact with the tumor epithelium.

In this study, we apply NanoString GeoMx digital spatial profiling (DSP) to TNBC across the three clinical states, nonmetastatic (*n* = 6), metastatic primary (*n* = 13), and matched LN metastases (*n* = 10), with three additional unmatched primary (total primary profiled = 19; total specimens including LNs = 29). Overlay of reference-based immune deconvolution, TNBC subtypes adapted to spatial regions, and spatially aware T-cell phenotype, on the region of interest (ROI) of nonmetastatic, metastatic primary, and matched LN metastases, revealed (i) a nonmetastatic state that is transcriptionally distinct from metastatic state, (ii) heightened T- and B-cell signals in metastatic primary, and (iii) immune-cold, macrophage-skewed niches in LNs. By linking subtype identity [BL1/BL2/IM/M/LAR/unspecified (UNS)] to immune–spatial classes (inflamed, excluded, and ignored), we reveal clinically relevant plasticity, including frequent switching to UNS/IM with nodal spread that refines biological understanding of TNBC progression.

## Materials and Methods

### Selection of patients

The study was done on a breast cancer tissue microarray (TMA; Novus Biologicals) that included 6 nonmetastatic and 13 primary breast tumors, 10 of which had matched metastatic LN samples. All samples were collected prior to systemic therapy. Written informed consent was obtained from all participants prior to sample collection. This study was conducted in accordance with the Declaration of Helsinki and approved by the institutional ethical review board under HPRC #011723: “Spatial transcriptomic analysis of patients with breast cancer.” The research project is classified “EXEMPT” and was approved for implementation consistent with 45 CFR 46.104 (d)4.ii.

### Performing spatial transcriptomics

We selected distinct tumor epithelium and stroma ROIs per tumor core. For each tumor core, a 300 μmol/L ROI region was selected, attempting to acquire the largest percentage of tumor cells from each tumor core section. The cellularity of a typical core averaged 1,200 cells. Each ROI was segmented into tumor core and stromal region based on fluorescently staining utilizing pan-cytokeratin (epithelium marker), *CD45* (pan-lymphocyte), *CD68* (pan-macrophage), and *SYTO83* for cell nuclei. RNA sequencing (RNA-seq) using the NanoString GeoMx DSP platform yielded expression data for the whole transcriptome across 57 ROIs identified based on immunostaining patterns for the morphologic markers. None of the ROIs was sequenced below 50% of saturation. The sequencing data were normalized using the third-quartile expression (Q3), and the 75% quantile-scaled data were used for all subsequent analyses.

For spatial transcriptomic analyses, we selected distinct tumor epithelium and stroma ROIs per tumor core. Data from one or two ROIs were captured per patient, and segmentation was performed on each ROI to distinguish the stromal and tumor compartments. For each tumor core, a 300 μmol/L region of ROI was selected, attempting to acquire the largest percentage of tumor cells from each tumor core section. The cellularity of a typical core averaged 1,200 cells. Each ROI was segmented into tumor core and stromal region based on fluorescently staining utilizing pan-cytokeratin (epithelium marker), *CD45* (pan-lymphocyte), *CD68* (pan-macrophage), and *SYTO83* for cell nuclei. After ultraviolet illumination, barcodes were collected in 96-well plates and dried for 1 hour at 65°C. Photocleaved oligos were resuspended in 10 μL nuclease-free water and hybridized to GeoMX Hyb codes at 65°C for 18 hours. Samples were finally pooled and processed on the nCounter MAX system (NanoString). nCounter counts were converted to digital count conversion file using the NanoString’s GeoMx NGS pipeline (v2.1) and imported back in the GeoMx DSP platform to generate an expression count matrix for the whole transcriptome. After ROI quality control according to NanoString’s recommendations, principal component analysis (PCA) was performed to eliminate potential outliers.

### Spatial T-cell phenotype determination

Hammerl and colleagues (10) defined three spatial immunophenotypes based on the distribution of CD8^+^ T cells: excluded (CD8^+^ T cells predominantly at the tumor border, absent from the center), ignored (minimal CD8^+^ T cells in both the border and center), and inflamed (CD8^+^ T cells evenly distributed across both regions). We applied this classification using expression level of CD8^+^ T cell–related markers (*CD3E*, *CD68*, *CD8A*, *CD8B*, *MS4A1*, *NCAM1*/*CD8*, *CD3*, *CD20*, and *CD56*) in the tumor and stromal compartments. We first classified the high/low level the level of these markers in all tumors and stroma and then identified the spatial T-cell phenotypes according to the combined expression patterns.

### Detection of differentially expressed genes

Area of illumination (AOI) raw counts were normalized, differential gene expression was tested using the Deseq2 package (v3.14; ref. 11), and *P* values adjusted using the Benjamini–Hochberg correction. Genes with a q value ≤ 0.05 were identified as differentially expressed genes (DEG). Functional enrichment of DEGs was performed through Enrichr (12). Significant Gene Ontology terms and Kyoto Encyclopedia of Genes and Genomes pathways were identified with *P* value < 0.05. Furthermore, gene set enrichment analysis (GSEA; ref. 13) was conducted to determine gene sets that were significantly different between primary metastatic tumors and nonmetastatic tumors. The H (h.all.v6.2.symbols.gmt) subcollection from the Molecular Signatures Database (http://software.broadinstitute.org/gsea/msigdb/index.jsp) served as the reference for GSEA, with a normalized enrichment score and false discovery rate denoting statistical relevance. The gene expression signatures were estimated using the signifinder package (14). Heatmaps were generated using the pheatmap R package (v1.0.12; ref. 15) after averaging the gene expression of the indicated AOIs per patient.

### Mixed tumor and immune cell deconvolution

Based on gene expression, abundances of infiltrating immune cells were estimated using CYBERSORTx (16). Mixed cell deconvolution was analyzed by the SpatialDecon package (17). Significant differences in immune cells between tumor groups were defined at the cutoff *P* value = 0.05 using the Mann–Whitney U test.

### PAM50 and TNBC subtyping analyses

For PAM50 subtypes, classification of samples by PAM50 subtypes was performed using the Genefu R package (version 1.16.0; ref. 18). For TNBC subtypes, patients with TNBC were labeled according to “TNBC type” via the web-based tool (http://cbc.mc.vanderbilt.edu/tnbc/; ref. 19).

### Survival analyses

The “Survival” package (v3.3-1) was used to build a standard survival object. Overall survival (OS) was defined as the time from the start of treatment until death or the last patient contact. To evaluate this, Kaplan–Meier survival curves were generated. Survival curves for different groups were compared, and the log-rank test was used to evaluate significant differences in survival durations (*P* value < 0.05).

### Statistics

Spearman’s correlation analyses were performed for assessment of correlations. Nonparametric Mann–Whitney U tests were applied for comparisons between two different groups of patients. Ordinary one-way ANOVA was performed for comparisons of all three groups. Statistical analyses were conducted using R Studio (v1.2.1335) and R (v3.6.1). Specific R packages and methods used in each step are described in the preceding sections.

## Results

### Cohort overview

**Figure 1.**
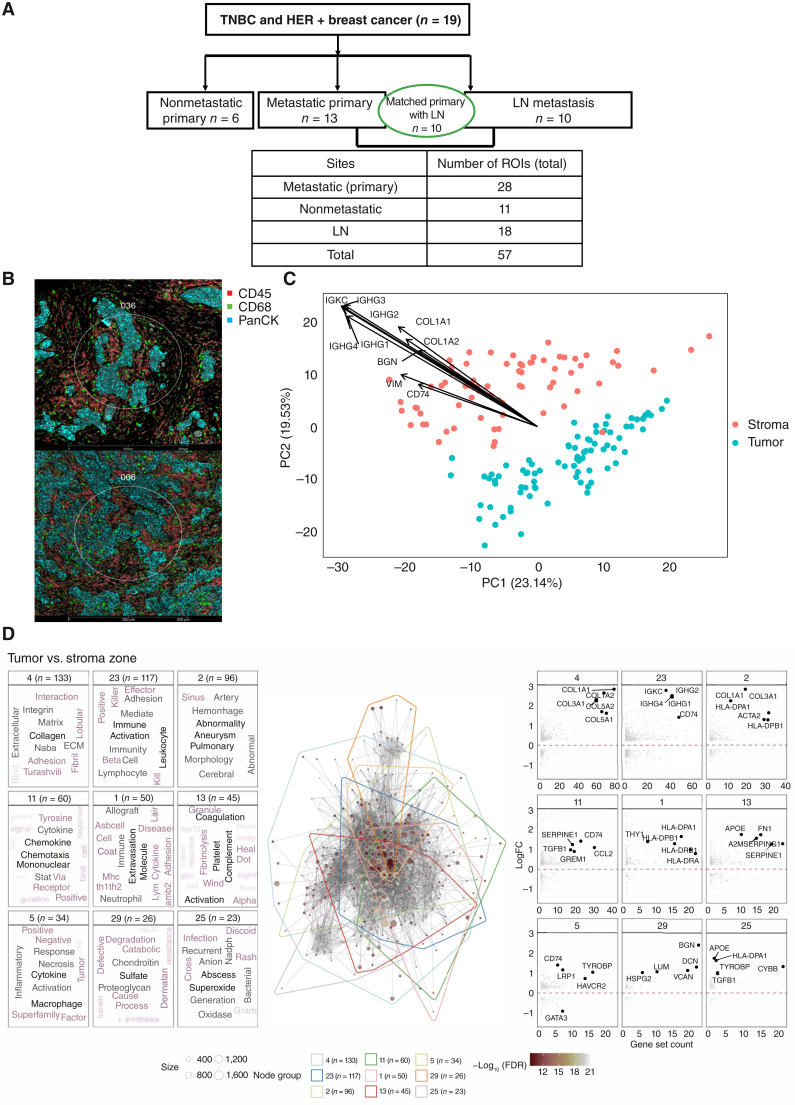
Transcriptomic difference between tumor stroma and tumor core. **A,** Overview of the data sources used in the study and transcriptomic difference between tumor core and stroma compartments. Nonmetastatic: Breast tumor without metastasis. Primary metastatic: Breast tumors with metastasis. LN metastasis: LN tumors metastasized from breast tumor. **B,** Representative TMA core of two ROIs. The white circle delineates ROIs selected for transcriptomic profiling. Cyan: epithelial cells visualized by pan-cytokeratin. Red: CD45-positive immune cells. Green: CD68-positive macrophages. **C,** PCA plot of tumor core and stroma samples. **D,** A typical vissE analysis of DEGs and produces three plots: a word-cloud, a network, and a gene statistic plot. The word-cloud plot performs a text-mining analysis to automatically annotate gene set clusters (top 9 in this case, ordered by cluster size and the −log_10_ of the FDR). The network plot visualizes gene sets as a network in which nodes are gene sets and edges connect gene sets that have genes in common; Gene statistic plots visualize a gene-specific statistic (a log fold change in this case) for all genes that belong to gene sets in the cluster against the number of gene sets that gene belongs to.

We utilized a TMA that consisted of 29 tumor cores from 19 patients (Fig. 1A). The median age was 44 years (range, 34–66 years old), with all tumors diagnosed with stage II or III disease (Table 1; Supplementary Data). We analyzed a total of 57 ROIs, comprising 28 ROIs from primary metastatic tumors (LN-positive), 18 ROIs from paired LN samples, and 11 ROIs from nonmetastatic primary tumors (LN-negative). Metastatic tumors were a mix of patients with HER2 positivity and TNBC (4 HER2+ and 6 TNBC), whereas all six nonmetastatic tumors were TNBC.

**Table 1.** Clinical characteristics of the analyzed cohort (*N* = 19).

​	*N*	% or range
Median age/age range	44	34–66
Median tumor size/tumor size range (cm)	3.5	2.5–10
Died in cohort?	​	​
Yes	3	15.79%
Recurrent in cohort?	​	​
Yes	13	68.42%
Stage	​	​
II	10	52.63%
III	9	47.37%
TP53 mutated in cohort?	​	​
Yes	9	47.37%

We utilized the GeoMx morphologic markers (pan-cytokeratin) to identify epithelial cells within each ROI, henceforth referred to as the PanCK+ AOI/segment, and CD45^+^ and CD68 antibodies to capture the immune-rich region of the ROI, referred to as the PanCK− AOI/segment (Fig. 1B; Supplementary Fig. S1A). The PanCK+ and PanCK− AOI/segments showed separation, revealing that the PanCK− stromal AOIs exhibited a distinct cluster compared with the PanCK+ tumor AOIs (Fig. 1C). Within the stroma, immune-related genes such as *IGKC*, *IGHG3*, and *CD74*-markers, which play a functional role in antigen processing and immune engagement, were highly expressed compared with tumor cells (Supplementary Fig. S1B). However, comparison of DEGs within the stromal compartment revealed a mixture of macrophage-related markers (*CD68*, *C1QC*, *CCL2*, and *SPP1*) and extracellular matrix (ECM) remodeling genes (*COL1A1*, *COL5A2*, *VIM*, *SPARC*, and *FBN1*; Supplementary Fig. S1B; ref. 20). GSEA revealed that there is an upregulation of genes associated with cell–cell interaction and immune-related pathways in the stroma (Fig. 1D). The transcriptional diversity within the stroma likely reflects a mixed tumor–immune cell population, indicating the presence of tumor-associated macrophages, which may contribute to the formation of an immunosuppressive, lymphocyte-excluded environment. Additionally, overexpression of ECM remodeling genes such as *VIM*, *COL1A1*, and *FBN1* suggests increased tumor stiffness and promotion of epithelial-to-mesenchymal transition (EMT).

### Characterizing the epithelial and nonepithelial cells in different clinical states in TNBC

To explore intraumoral and intertumoral heterogeneity between nonmetastatic and primary that have paired LN metastases, we performed a multitiered transcriptomic and immune landscape analysis by integrating PCA, gene expression profiling, pathway enrichment, and two complementary approaches for immune cell deconvolution: a curated cell-type signature–based method and CIBERSORTx (16).

**Figure 2.**
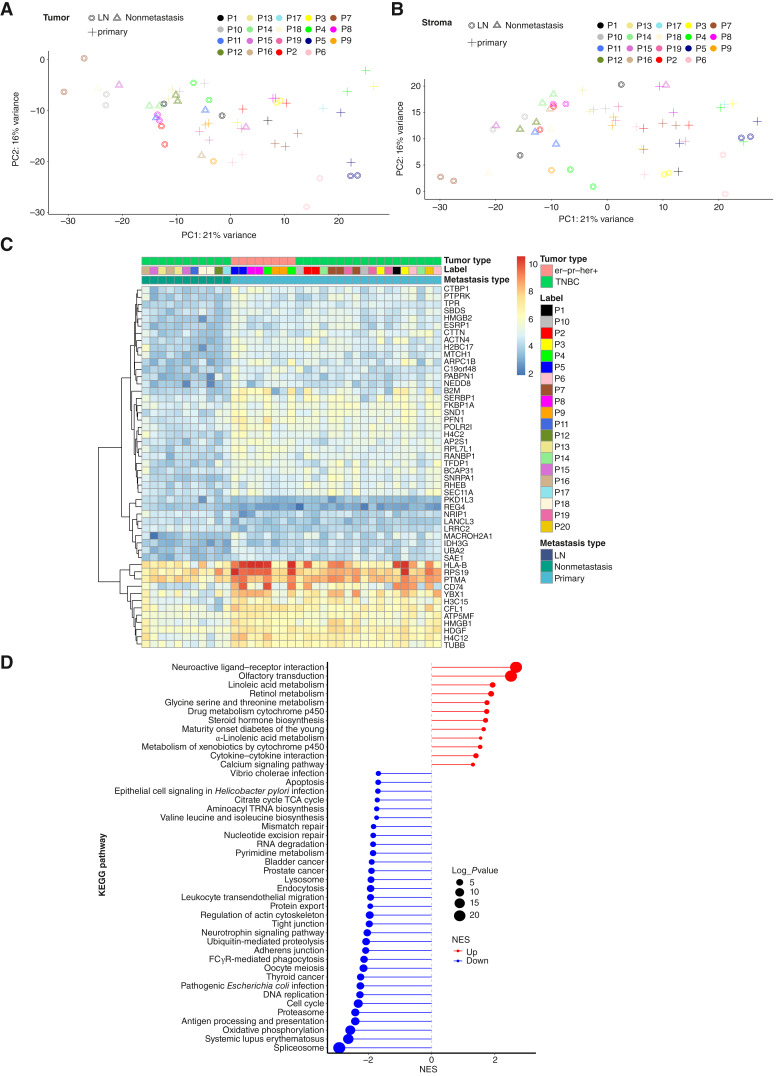
Transcriptional heterogeneity between primary metastatic and nonmetastatic tumors. **A,** PCA plot showing the distribution of tumor samples. **B,** PCA plot showing the distribution of stroma samples. **C,** Heatmap showing the top DEGs between primary metastatic tumors and nonmetastatic tumors. **D,** Enriched pathways analysis of DEGs identified between primary metastatic and nonmetastatic tumors. KEGG, Kyoto Encyclopedia of Genes and Genomes; NES, normalized enrichment score.

We first applied PCA to spatially segmented AOIs to assess transcriptomic variation across the three clinical states and analyzed separately for tumor epithelium and stroma (Supplementary Fig. S2A). PC1 (21% variance) separated primary tumors from LN and nonmetastatic samples, which largely overlapped, with only a few LN shifting toward the primary tumors (Fig. 2A). PCA plots revealed no clear separation between HER2+ and triple-negative tumors, despite the mixed composition of the cohort, implying that the HER2+ subset was transcriptionally similar to TNBC. PC2 (16% variance) captured interpatient heterogeneity, showing vertical spread within groups; nonmetastatic samples displayed strong separation along PC2, whereas primary tumors remained more central.

In the stromal PCA (Fig. 2B), the metastatic signal is represented along the left PC1 and primary shows a rightward (positive PC1) shift, and a subset of LN extends into the same positive PC1 tail. PC2 reflects the nonmetastatic signal, in which it displays a strong vertical separation along the PC2 axis. The separation between nonmetastatic and primary tumors along PC2 is clearer in the stroma compared with the PanCK+ epithelial cells. Overall, the groups that overlap between the stroma is greater than in the epithelial compartments, indicating that patient-specific differences such as genetic, immune, or other factors outweigh the effect of the tumor on stromal cells.

### Nonmetastatic TNBC is transcriptionally distinct from metastatic primary TNBC

The PCA of epithelial cells in nonmetastatic and metastatic AOIs showed a clear separation, suggesting distinct biological programs underlying the separation. In the heatmap (Supplementary Fig. S2B), two coherent epithelial modules align with metastatic status. Nonmetastatic upregulated genes were associated with luminal/secretory pathways that included *TFF3*, *MUCL1*, *PSCA*, *APOD*, and multiple ribosomal subunits (e.g., *RPL23* and *RPL7L1*; Fig. 2C; Supplementary Fig. S2C), whereas primary metastatic tumors had highly expressed immune presentation and interferon (IFN)-responsive genes, characterized by *HLA-A*/*HLA-B*, *B2M*, and *CD74* (class-II chaperone) and often accompanied by cytoskeletal/adhesion elements such as *ACTN4*, *ARPC1B*, *CTTN*, and *CFL1* (Fig. 2C; Supplementary Fig. S2C). The functional enrichment analysis also showed that nonmetastatic tumors activated multiple metabolic pathways, including retinol/steroid metabolism, linoleic acid metabolism, and glycine, serine, and threonine metabolism. In contrast, these nonmetastatic tumors exhibited downregulated genes that were more varied and enriched for antigen presentation, proteosomes, oxidative phosphorylation, and DNA replication/cell cycle (Fig. 2D).

TNBC can be broadly classified into several subtypes based on gene expression and other molecular characteristics (2), which is critical for guiding precision therapeutic strategies. To further characterize the biological and clinical diversity of tumors across anatomic sites, we performed Lehmann TNBC subtyping to classify each sample into molecular subtypes: BL1, BL2, IM, M, MSL, LAR, or UNS (Supplementary Fig. S3).

**Figure 3.**
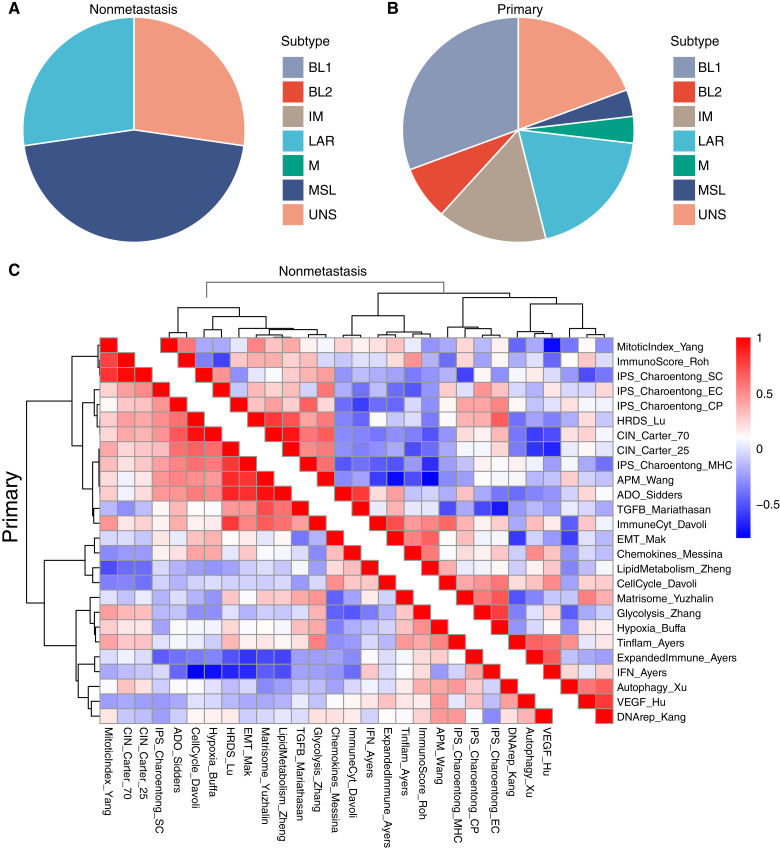
Disparities of subtype distribution and gene expression signature patterns between primary metastatic and nonmetastatic tumors. **A** and **B,** Pie chart showing the distribution of TNBC subtypes in primary nonmetastatic (**A**) and metastatic tumors (**B**). **C,** Heatmaps showing correlations among gene expressional signatures in primary nonmetastatic tumors (right) and metastatic tumors (left).

We next computed TNBC molecular subtype (Lehmann classification) on these two clinical states. Among the nonmetastatic epithelial AOIs, we identified three predominant subtypes: MSL was the most common (46%), followed by UNS and LAR, which were equally represented (27% each; Fig. 3A). Among the primary metastatic samples, the BL1 subtype was the most prevalent (31%), followed by LAR and UNS at 19% each, IM at 15%, and smaller proportions of BL2, M, and MSL subtypes (Fig. 3B). The variation in subtype frequency between nonmetastatic and metastatic AOIs mirrors the differential activation of the core molecular pathways that constitute the Lehmann classification.

To investigate the interplay of core oncogenic pathways, we used Signifinder (bioRxiv 2023.03.07.530940), a spatially informed analytic framework that moves beyond traditional GSEAs by accounting for tissue heterogeneity. These signatures represented different biological pathways. It includes proliferation and genomic instability, IFN and immune-relative activity, and metabolic stress programs. Using pairwise correlation matrices of hallmark gene expression signatures within the nonmetastatic and primary metastatic tumor cohorts, Signifinder ensured that observed patterns reflected profound transcriptional rewiring between the two cohorts than spatial contamination from adjacent cell types (Fig. 3C).

In nonmetastatic primary tumors, the correlation structure was fragmented, with a pattern that chromosomal instability (CIN) signatures were exclusive correlated immune-related signatures, proliferation-associated programs, and metabolic, angiogenic programs (Fig. 3C). The correlation of topology in primary metastatic tumors was fundamentally altered. A consolidated and tightly covarying immune/IFN-inflamed cluster emerged as a dominant feature. Notably, this integrated immune cluster exhibited strong mutually exclusivity (deep blue off-diagonal) with CIN and DNA replication signatures, revealing a transcriptional trade-off between a proliferative phenotype and an immune-inflamed microenvironment in metastasis-capable tumors (Fig. 3C).

### Subtype divergence in LN metastasis

**Figure 4.**
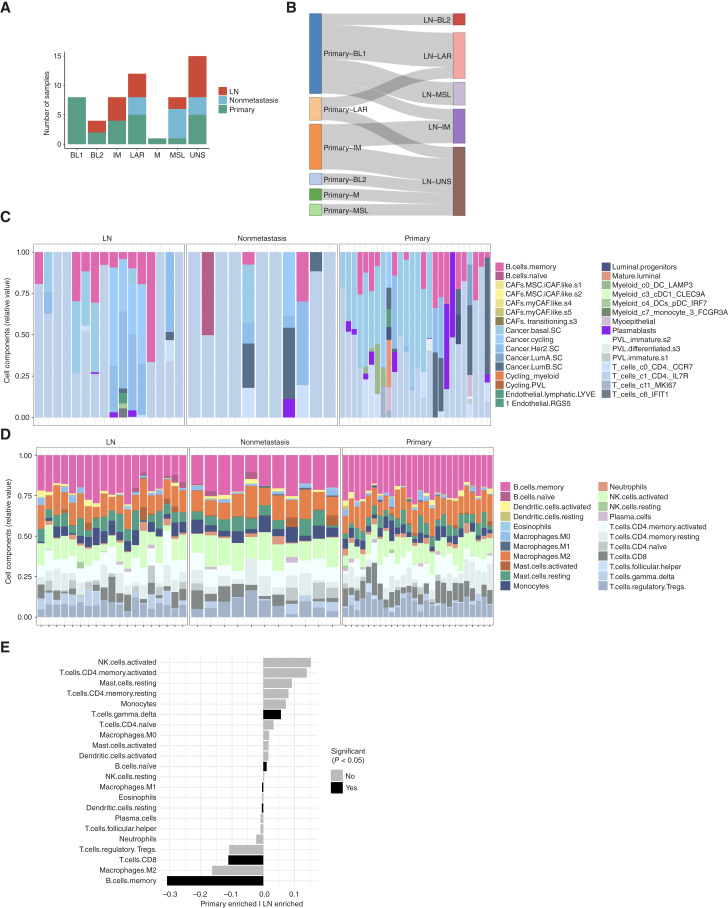
Subtype reprogramming in LN metastasis. **A,** Stacked bar plot showing the frequencies of TNBC subtypes in primary metastatic, primary nonmetastatic, and LN metastases. **B,** Sankey plot showing subtype transitions from primary tumor to matched LN metastases. **C,** Stacked bar plot showing mixed cell populations estimated using SpatialDecon. **D,** Stacked bar plot depicting infiltrating immune cells inferred by CYBERSORTx. **E,** Bar plot showing immune cell populations enriched in primary tumors (left) and LN metastases (right). Statistical differences across subtypes were assessed using Wilcoxon the rank-sum test when comparing each subtype with each of the others.

We observed discordant subtypes across primary and matched LNs from the same patient, reflecting site-level heterogeneity. LN AOIs showed separation from the primary metastatic AOIs but overlapped considerably with the nonmetastatic AOIs. LN metastases were predominantly classified as UNS, IM, and LAR (Fig. 4A). Analysis of paired primary and metastatic lesions revealed a substantial subtype divergence in LN metastases, as shown by the Sankey diagram (Fig. 4B) Primary tumors that were predominantly of the BL1 subtype exhibited LAR or MSL phenotypes in their matched LN metastases (71%), whereas smaller fractions were of BL2 or IM states (Fig. 4B), suggesting a selective advantage or adaptive mechanism favoring these subtypes in the LN microenvironment. In LN metastases that had the LAR subtype, the corresponding primary tumors were restricted to either the LAR or IM subtype. However, for primary IM tumors, the metastatic sites were phenotypically heterogeneous, with half retaining the IM signature and half being classified as UNS. Studies have reported high concordance of molecular subtype between primary and metastatic TNBCs, particularly for LAR, IM, and BL1, with instability largely confined to the MES subtype (21–23). In contrast, in matched primary–LN pairs, we quantify a distinct, site-specific reclassification pattern in which BL1 primary most frequently classify as LAR or MSL in LNs, accompanied by an enrichment of UNS subtype in LN metastasis. To our knowledge, this has not previously been identified in paired TNBC cohorts.

### Distinct cell-type and immune repertoires characterize nonmetastatic, metastatic, and LN metastatic clinical states in TNBC

In order to delineate how cellular architecture and immune repertoires differ across nonmetastatic primary TNBC, LN metastases, and distant metastases, we used two distinct methods for the profiling their landscape. First, to resolve the cellular architecture, we performed spatial deconvolution—a computational method that quantifies abundances of cell type, providing essential cellular context that spot-level expression data alone cannot reveal (17). Following, we applied CIBERSORTx (16) to estimate the relative abundance of canonical immune cell populations.

Comprehensive spatial deconvolution of our cohort revealed fundamentally distinct cellular ecosystems across sites. In nonmetastatic primary tumors, the epithelial compartment was predominantly composed of luminal progenitor and mature luminal cells, with only a minor population of cancer cells. A small cycling fraction and occasional myoepithelial cells were also observed (Fig. 4C). Their stromal landscape comprised of PVL immature subsets s1 and s2, a PVL differentiated subset s3, and endothelial (RGS5^+^ and LYVE1^+^) cells. The immune microenvironment demonstrated an innate–dominant composition, featuring relatively abundant population of activated NK cells, resting NK cells, and neutrophils alongside a modest population of (naïve and memory) B cells and plasma blasts (Fig. 4D). Additionally, low levels of myeloid dendritic cell (DC; LAMP3^+^, CLEC9A^+^, IRF7^+^, and FCGR3A^+^) subsets and very limited number of CD4^+^ T (CCR7^+^ and IL7R^+^), proliferating T (MKI67^+^), and IFN-stimulated T (IFIT1^+^) cells were present (Fig. 4C).

In contrast, metastatic primary tumors exhibited the greatest cellular diversity (Fig. 4C). The epithelial compartment composed of multiple states: luminal progenitors; mature luminal, luminal A, and luminal B cancer cells; basal cancer cells; cycling cancer cells; and patchy myoepithelial cells. Stromal complexity was heightened with multiple PVL programs and both endothelial cell (RGS5^+^ and LYVE1^+^) classes. The immune compartment was most expansive, showing robust expansion of CD4^+^ (CCR7^+^ and IL7R^+^), CD8^+^, proliferating (MKI67^+^), and IFN-stimulated (IFIT1^+^) T cells. This was complemented by significant (memory and naïve) B-cell population, plasmablasts, and myeloid DC subsets (LAMP3^+^, CLEC9A^+^, and_IRF7^+^), alongside myeloid (FCGR3A^+^) monocytes.

LN metastases demonstrated a contracted ecosystem dominated by epithelial components, primarily luminal progenitors and mature luminal cells with substantial fraction of cycling cancer cells. Stromal diversity was reduced with diminished myoepithelial presence, simplified PVL programs, and decreased endothelial heterogeneity. The immune microenvironment was characterized by persistent memory B cells (outnumbering naïve B cells), CD4^+^ T cells, and small plasmablast pockets. Low myeloid DC subsets (CLEC9A^+^) were presented (mean = 0.018). Although some CD4^+^ T-cell subsets (CCR7^+^ and IL7R^+^) were present, cytotoxic CD8^+^, proliferating T (MKI67^+^, mean = 0.009), and IFN-stimulated T (IFIT1^+^, mean = 0.0034) cells were notably scarce. This cellular architecture is consistent with an immune-excluded nodal niche that supports metastatic colonization despite detectable immune presence (Fig. 4C; Supplementary Fig. S4A).

Stromal signatures were derived by staining stroma for CD68 and CD45 markers. However, to estimate the relative abundance of only canonical immune cell populations, we applied CIBERSORTx (Fig. 4D; Supplementary Fig. S4B). Deconvolution of stromal ROIs revealed that all groups were dominated by B-lineage signal (memory/naïve B cells), but the balance of other lineages shifted by site. Nonmetastatic samples displayed a more innate–skewed profile, with larger bands for (activated/resting) NK cells and neutrophils across many samples and the smaller T-cell compartment than primary tumors. In primary metastatic tumors, the immune infiltration is highly heterogeneous, with memory B cells accounting for the largest fraction with substantial contributions from both naïve and plasma B cells (*P* = 0.03). We also observed a mix of CD4^+^ T-cell subsets [resting, activated memory, follicular helper, and regulatory T cells (Treg)], CD8^+^ T cells (*P* = 0.018), as well as significant presence of both M1‐ (*P* = 0.018) and M2‐polarized macrophages alongside monocytes, mast cells, and activated DCs (*P* = 0.004). By contrast, LN metastases show a striking shift toward a more immunosuppressive profile comprised of a myeloid‐ and B cell–dominated milieu with abundance of memory B cells along with increase in Treg (*P* = 0.46) and neutrophil (*P* = 0.18) populations. However, naïve B cells and plasma cells diminished, CD8^+^ T cells contracted, and macrophage polarization skews further toward the M2 phenotype (*P* = 0.4; Fig. 4E). The increased expression of immune checkpoints genes that included *CD274* (*PD-L1*), *CTLA4*, and *IDO1* was seen in the LN TME as compared with nonmetastatic and matched primary metastatic tumors (Supplementary Fig. S4C). Thus, the metastatic niche in the LN is characterized by a loss of diverse antitumor lymphocyte subsets and an enrichment of immunoregulatory macrophages and memory B cells compared with the more balanced, effector‐rich landscape of the primary tumor.

Collectively, these results demonstrate that tumor heterogeneity extends beyond gene expression profiles to include the immunologic microenvironment (Supplementary Fig. S4A and S4B). Aligning with differential expression of several immune checkpoint genes between breast and LN tumors, the consistency of findings across two independent immune profiling methods, a tumor-specific signature matrix and CIBERSORTx—strengthens the evidence that metastatic tumor environments are immunologically distinct, likely reflecting adaptive changes to the LN niche and potential mechanisms of immune escape.

### Clinical state–integrated, subtype-resolved correlation of immune, stromal, and proliferative programs

The three clinical states exhibited overlapping subtypes as shown in (Fig. 4A). The PCA of all tumors revealed that BL1 and IM subtypes from primary tumors shared similar transcriptomic profiles, whereas MSL, LAR, and UNS subtypes displayed greater dispersion, suggesting increased molecular variability or instability (Supplementary Fig. S5A). Importantly, LN-associated subtypes were distant from their corresponding primary counterparts, suggesting that LN metastases often undergo subtype divergence accompanied by transcriptional reprogramming.

**Figure 5.**
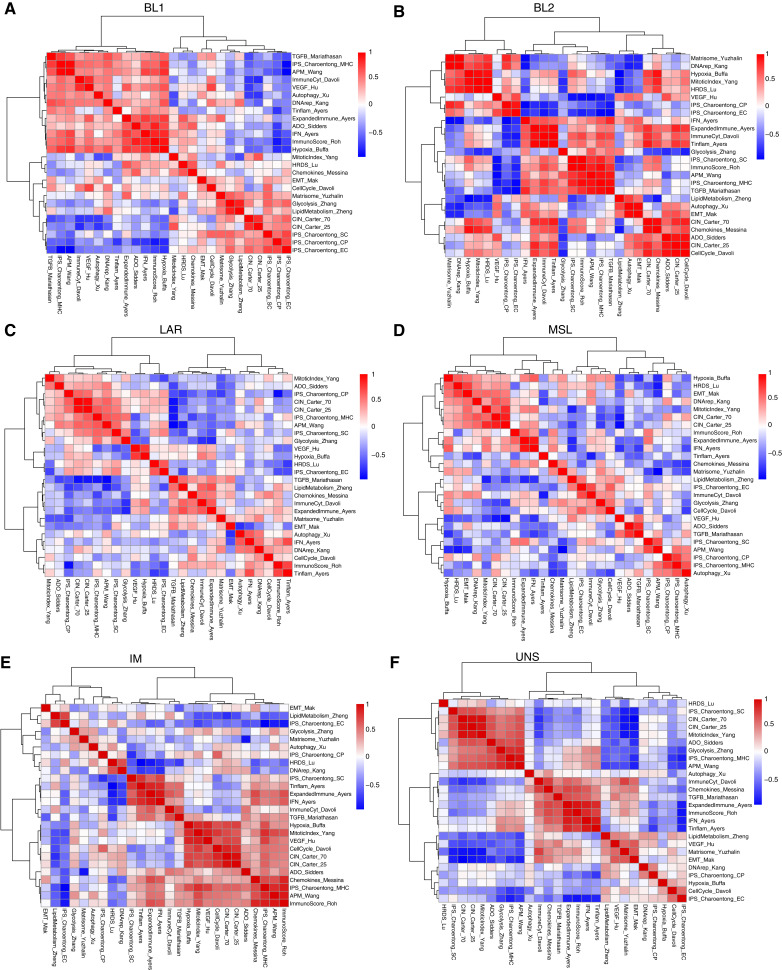
TNBC subtyping reveals independent gene expression signatures. **A–F,** Heatmaps showing the correlations among gene expressional signatures within BL1 (**A**), BL2 (**B**), LAR (**C**), MSL (**D**), IM (**E**), and UNS (**F**) subtypes.

To further characterize the biological programs underlying each TNBC subtype, we examined the expressional signatures specific to each subtype. Pairwise correlation of hallmark pathway signatures within each molecular subtype revealed distinct and biologically coherent network structures (Fig. 5A–F). In the BL1 and BL2 subgroups, immune‐related modules showed a strong positive correlation with a consolidated immune-inflamed program. In contrast, proliferative and genomic instability signatures, such as CIN and DNA replication, were largely anti-correlated with immune activity (Fig. 5A and B). These patterns reveal a transcriptional trade-off between proliferative and immune-inflamed states within BL1 and BL2 tumors (Fig. 5A and B). This pattern was attenuated in the LAR subgroup, which shows immune programs, IFN, cytotoxicity, chemokines, and the IPS subscores EC, CP, and SC, which are mutually correlated and inversely related to proliferation and genomic instability measures (Fig. 5C). MSL tumors displayed a consolidated correlation structure proliferation, genomic instability metrics, and stromal–EMT/angiogenic programs. Metabolic stress signatures and autophagy were negatively correlated with all immune programs in this subgroup (Fig. 5D), which suggests that this “proliferative and unstable” strategy is a distant oncogenic program. In the IM subgroup (Fig. 5E), immune‐related pathways were strongly correlated with autophagy, TGFβ signaling, and CIN, while inversely correlating with homologous recombination deficiency and DNA replication. This pattern implies that there are other mechanisms are linked to genomic changes in the immune-activated state of IM tumors. Finally, the UNS subtype exhibited a weak and fragmented correlation structure, with immune-related signatures, proliferation-associated programs, and cell cycle exclusive correlated with each other (Fig. 5F). This indicates that UNS tumors lack a dominant transcriptional program and exhibit heterogeneous biological states.

Importantly, subtype classification also revealed different survival outcomes (Supplementary Fig. S5B; *P* < 0.05). BL2 and MSL showed the best survival, BL1 was intermediate, whereas IM and UNS exhibited the poorest survival. This suggests that tumors classified as IM or UNS, which were more common in LN metastases, may be associated with poorer prognosis and more aggressive phenotypes. Collectively, these findings suggest that metastatic progression is often accompanied by transcriptional reprogramming toward less defined or more aggressive molecular subtypes.

### Molecular subtypes are distinctly associated with different T-cell phenotypes

**Figure 6.**
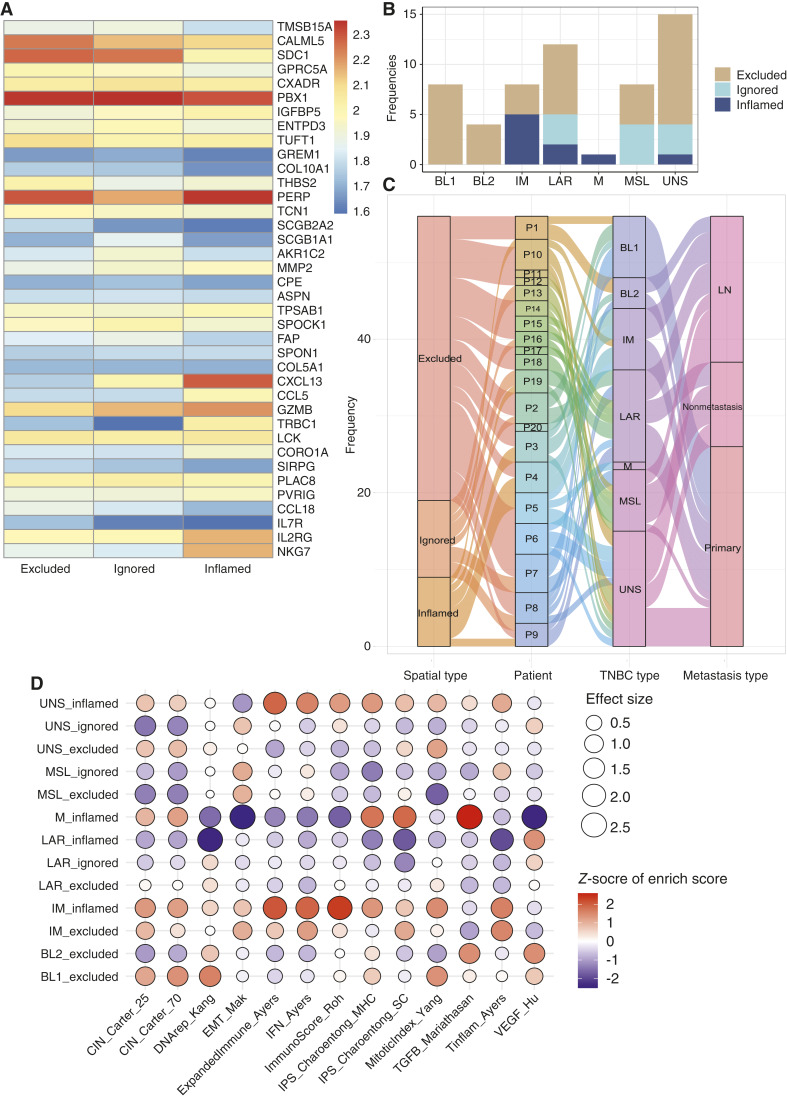
Spatial transcriptomics reveals divergence in immune phenotype and functional programs within the same TNBC subtypes. **A,** Heatmaps showing the average expression level of selected markers across three spatial phenotypes (excluded, ignored, and inflamed). **B,** Stacked bar plot showing the distribution of TNBC subtypes across the three spatial phenotypes. **C,** Sankey plot showing the distribution of patient characteristics across clinical and molecular categories. **D,** Bubble plot showing differences in gene expressional signatures among ISMS subgroups.

Using an established framework for spatial–immune categorization (10), we assigned each tumor to an inflamed, excluded, or ignored phenotype category based on a predefined gene expression classifier (Fig. 6A). CIBERSORTx deconvolution independently validated these classifications, confirming significant variations in immune cell composition across the distinct phenotypes (Supplementary Fig. S6A). Inflamed tumors exhibited high expression of key genes (*GZMB*, *TRBC1*, *LCK*, *CXCL13*, *CCL5*, *IL7R*, and *NKG7*) involved in responsible for cytotoxic T-cell activity, immune recruitment, and T-cell receptor signaling indicating higher NK-cell and T-cell populations (Fig. 6A; Supplementary Fig. S6A; refs. 24–27). By contrast, excluded tumors exhibited elevated expression of stromal and ECM-related genes like*TMSB15A*, *CALML5*, *SDC1*, *GREM1*, *FAP*, and *COL10A1* (28–31), suggesting that immune cells were restricted to the tumor periphery within fibrotic or desmoplastic stroma, limiting infiltration into tumor parenchyma. The ignored phenotype, intermediate in its immune gene expression profile, displays lower expression of both cytotoxic markers and exclusion-related genes (*CCL18*, *IL7R*, *IL2RG*, *CXCL13*, and *CCL5*). These tumors may represent immune desert states, in which immune evasion occurs due to lack of immune activation or tumor antigen presentation.

Integration of spatial immune phenotypes with molecular subtypes revealed distinct associations, with specific distributions (Fig. 6B). BL1 tumors were exclusively found in primary sites and uniformly excluded (Fig. 6B and C). Similarly, BL2 tumors were also excluded, primarily located in primary and LN metastases. In contrast, the IM subtype was bifurcated into excluded and inflamed states, which are also from primary and LN metastases.

Furthermore, the LAR subtype exhibited broader heterogeneity, spanning all three immune phenotypes across all anatomic sites. Inflamed LAR tumors were enriched in primary sites, whereas excluded/ignored LAR tumors were observed in both LN and nonmetastatic sites. MSL tumors were distributed into excluded and ignored, sparsely distributed across LN and nonmetastatic sites. Lastly, the UNS subtype was predominantly excluded and most frequent in LN metastases, with smaller populations of ignored tumors and rare inflamed instances observed across other groups.

### Subtype-specific T-cell phenotype and associated pathway signatures classify tumors into immuno-spatial molecular subtypes

Overlaying hallmark programs on the subtype associated with the T-cell spatial phenotype defined immuno-spatial molecular subtypes (ISMS) that expose biology not visible in subtype alone. We observed distinct pathway enrichment patterns associated with specific subtypes across the six primary‐to‐LN TNBC subtype–immune phenotype combinations (Fig. 6D). Within inflamed classes, IM_inflamed showed the strongest IFN–MHC–Immunoscore with minimal EMT/TGFβ/VEGF (a *bona fide* T cell–inflamed state), M_inflamed retained immune activation and concurrently elevated proliferation/CIN (“hot-but-mesenchymal”), and UNS_inflamed showed moderate immune activation with largely neutral stroma/proliferation. Among excluded classes, MSL_excluded and IM_excluded were defined by high EMT/TGFβ/VEGF with suppressed IFN/MHC (canonical exclusion), LAR_excluded coupled these barriers to higher proliferation/CIN, BL1_excluded and BL2_excluded combined strong proliferation/CIN with low IFN/MHC and upregulated EMT/TGFβ, and UNS_excluded showed the same triad at intermediate effect sizes. Ignored classes (LAR_ignored, MSL_ignored, and UNS_ignored) were uniformly immune-cold without strong EMT/TGFβ, suggesting poor antigen/unprimed immune cells rather than barrier-mediated escape (Fig. 6D; Supplementary Fig. S6B–S6D). Notably, multiple ISMS expressional signatures were also observed in an independent public dataset (Supplementary Fig. S6E; ref. 7). Together, the ISMS classification emphasizes the integration of gene expression of subtypes with their spatial immune context and suggests that immune spatial phenotype adds critical biological context. We observe that key immunoregulatory checkpoint genes are upregulated in inflamed subtypes, whereas segregate strongly according to both subtype and spatial phenotype (Supplementary Fig. S6F–S6H). *CD274* (*PD‐L1*) expression is relatively uniform but shows a modest elevation in the inflamed tumors while elevated in multiple excluded phenotypes. *CTLA4* remains low across most excluded phenotypes, with an increase in IM_inflamed and MSL_ignored tumors. *TIM-3* (*HAVCR2*) showed intermediate levels in IM_inflamed and is lowest in all excluded states. IDO1 exhibits the largest dynamic range, with the highest levels in IM_excluded and UNS_inflamed tumors, which are markedly above the minimal expression seen in the excluded or ignored groups. IL10 is expressed broadly but is enriched in the excluded subsets. *PDCD1* (*PD-1*) also showed upregulation of inflamed but not subtype-inflamed subsets. Finally, *VTCN1* (*B7-H4*) is notably upregulated in the UNS_ignored and UNS_excluded groups and significantly exceeds levels in the inflamed states, which suggest that UNS_ignored is a distinct and potentially immunosuppressive niche within the “UNS” subtype (Supplementary Fig. S6F–S6H).

Together, these data showed that even within a single TNBC subtype, tumors adopt divergent immune‐checkpoint landscapes that are closely linked to their spatial T-cell phenotype. This highlights the functional importance of integrating molecular subtypes with immune spatial context. The ISMS framework therefore provides a clinically relevant tool to predict immune evasion and guide personalized immunotherapy strategies.

## Discussion

TNBC is a heterogenous disease comprising at least five molecular subtypes, each linked to unique clinical outcomes and differential responses to neoadjuvant therapy. Emerging studies have investigated breast cancer heterogeneity using spatially resolved transcriptomics, either independently or in combination with single-cell analyses (32–35). Recent studies applying spatial transcriptomics to TNBC have demonstrated intertumoral and intratumoral heterogeneity, with spatially distinct gene expression patterns and immune landscapes across tumor regions (7, 34). The arching goal is to understand the biology of TNBC in nonmetastatic and metastatic diseases.

In this study, we applied spatial transcriptomics to delineate the intrinsic transcriptome of tumor core and stromal compartments of the three clinical states of TNBC, nonmetastatic, primary metastatic tumors, and matched LN metastases. Dissection of the spatial transcriptomic regions/area of interest by subtypes, deconvolution of the immune components, hallmark gene signature correlation network, and T-cell phenotype classification offered new perspectives into the spatial dynamics of these three clinical states. As no significant differences in gene expression pattern or immune cell composition were observed after excluding HER2+ samples (Supplementary Fig. S6I–S6K), and ERBB2/HER2 expression was low in HER2+ cases (Supplementary Fig. S6L), we combined HER2+ and TNBC samples into a single cohort for downstream analyses. We demonstrate that primary metastatic tumors possess a distinct core molecular identity that is not contained in nonmetastatic tumors. Surprisingly, LN metastases shed this metastatic signature and revert to a molecular profile more similar to nonmetastatic, which is consistent the discordance of TNBC subtypes in primary and recurrent/metastatic tumors (36). TNBC clinical states are biologically discrete, each with its own transcriptional and immune ecology. We observe rewiring of hallmark signature pathways relationships in metastasis-bearing tumors, a tightly integrated IFN/immune module becomes mutually exclusive with CIN/DNA replication, revealing a strong immune–proliferation trade-off that is only weakly apparent in nonmetastatic disease. Metastatic competence in TNBC selects for tumors that solve one of two “objectives,” proliferative tumors cells with stromal cells suppressing or evading immunity, or a tumor that potentiates an active immune microenvironment at the cost of reduced tumor cells proliferation. The OS of patients with nonmetastatic tumors was higher than primary tumors which eventually became metastatic (Supplementary Fig. S6M). We show for the first time that within each TNBC subtype, pathway activities covary in distinct, coordinated patterns. These subtype-specific correlation maps explain why tumors with similar average pathway levels can nonetheless differ in behavior and treatment response.

Our paired primary–LN analyses show that the LN is not just a new location for tumors cells but rather it reprograms the tumor ecosystem. Nodal metastases are enriched for UNS/IM/LAR subtypes and harbor a contracted, immune-excluded niche with fewer cytotoxic and cycling T cells, more memory B cells, macrophages with an M2-like profile, and higher checkpoint expression. This configuration plausibly supports metastatic seeding and persistence despite the presence of immune cells. Our findings corroborate the transcriptional and immune landscape differences observed between primary and LN tumors and provide further mechanistic insight into the plasticity and prognostic relevance of TNBC subtype dynamics. Several primary tumors originally classified as BL1, IM, or LAR transitioned to MSL, LAR, or UNS subtypes in the LN metastases. This suggests that basal-like and IM tumors may lose their original transcriptional characteristics during dissemination and potentially acquire features associated with mesenchymal differentiation, androgen signaling, or transcriptional ambiguity after metastasis. The frequent occurrence of the UNS subtype as a terminal state in LN tumors indicates that metastases may adopt transcriptionally undefined or mixed profiles not captured by the canonical TNBC subtypes. Similarly, the emergence of the MSL and LAR subtypes from diverse primary origins supports a model of convergent evolution toward more aggressive or immune-silent phenotypes. Conversely, a subset of tumors, such as those originally classified as LAR, retained subtype stability across sites, suggesting that certain transcriptional programs remain conserved during metastatic progression.

Classifying tumors into excluded, ignored, and inflamed immune phenotypes revealed distinct microenvironment profiles that do not fully align with canonical TNBC subtypes. Subtypes such as BL1, BL2, and UNS predominantly exhibit immune-excluded profiles, whereas IM and M subtypes retained inflamed microenvironment, reflecting variable immune engagement across tumors. The LAR subtype showed the greatest heterogeneity, spanning all immune phenotypes. These findings underscore that immune states are dynamic properties shaped by tumor–microenvironment interactions rather than fixed features of molecular subtypes. Importantly, immune phenotype often diverged within a given molecular subtype, indicating that subtype alone does not capture the spatial complexity of immune regulation. The ISMS framework proposed here highlights how spatially resolved immune context complements gene expression–based subtyping to better reflect tumor behavior, immune evasion strategies, and therapeutic vulnerabilities.

Our integration of each molecular subtype on its T-cell spatial phenotype (inflamed, excluded, ignored, plus a mesenchymal-inflamed state) with gene signatures generated a new classification, coined “ISMS.” Our spatially resolved data reveal that tumors with the same subtype label can display different microenvironments, one immune-hot and responsive to checkpoint inhibition and another immune-excluded or inert. Without spatial context, these differences would be hidden, potentially correlated with misclassification of immunotherapeutic eligibility or resistance.

Despite these strengths, this study has several limitations. First, the sample size is relatively modest, which may limit the statistical power for detecting differences in outcomes and biological features across several subtype and ISMS categories, particularly in sparsely represented groups, and may affect the generalizability of our findings. Therefore, larger cohorts will be required to robustly establish the independent prognostic value of ISMS beyond conventional subtype and clinical variables. Second, our ROI selection was guided by CD45, CD68, and PanCK markers, with a focus on immune cell–enriched regions. Although this strategy enabled detailed characterization of tumor–immune interactions, it may introduce selection bias. Third, our analysis is limited to transcriptomic data and lacks integration with other molecular layers such as genomic (DNA), single-cell RNA-seq, proteomic, or epigenomic profiles, which could offer a more comprehensive understanding of the complex molecular landscape of breast tumors with LN metastasis. Future studies incorporating multi-omics approaches, including single-cell RNA-seq for TNBC subtype identification and multiplex immunofluorescence or imaging mass cytometry (IMC) data for spatial T-cell subtype identification, would further strengthen these findings. Lastly, due to the nature of the available data, we were unable to assess potential associations between molecular features and treatment responses, which would be critical for translating these findings into clinical applications.

## Supplementary Material

Supplementary Data Supplementary Tables

Supplementary Figure 1 ROIs selections and Differential gene expression analysis between tumor and stroma compartments.

Supplementary Figure 2 Transcriptional Heterogeneity of all nonmetastatic and metastatic primary tumors.

Supplementary Figure 4 Diverse immune cell proportions between primary tumors and LN metastases.

Supplementary Figure 5 Molecular heterogeneity across TNBC subtypes in two sites.

Supplementary Figure 6 Spatial transcriptomics reveals divergence in immune phenotype and functional programs within the same TNBC subtypes.

Supplementary Figure 3 Molecular heterogeneity across TNBC subtypes.

## Data Availability

The NanoString GeoMx data of patients with breast cancer, including the IMC data, have been deposited into Zenodo under the following URL: https://doi.org/10.5281/zenodo.17635504. The codes used to analyze the breast cancer subtypes data in this study were developed using R and Shell. The analysis code used in this study is deposited in GitHub under the link https://github.com/HuangTUSK/TNBC_spatialTranscriptomics and Code Ocean https://codeocean.com/capsule/4733243/tree/v1. All other data generated in this study are available upon request to the corresponding author.
