# Supplementary material for: TNBC Spatial Transcriptomic Analysis across Clinical States Reveals Subtype-Specific Networks and Immunosuppressive Niches
Source: Cancer Res Commun. 2026 May 29;6(5):1246–60. doi: 10.1158/2767-9764.CRC-25-0808 (PMC13245550; doi:10.1158/2767-9764.CRC-25-0808)
Supplement: Supplementary Figure 1 — ROIs selections and Differential gene expression analysis between tumor and stroma compartments. [file crc-25-0808_supplementary_figure_1_suppsf1.docx]

**Supplementary Figure 1.** ROIs selections and Differential gene expression analysis between tumor and stroma compartments. **A**: Tissue Microarray (TMA) core analyzed by multiplex immunofluorescence of ROIs. The white circle delineates ROIs used for transcriptomic sequencing. Cyan: Epithelial cells visualized by Pan-Cytokeratin. Red: CD45 positive cells. Green: CD68 positive cells. **B**: Volcano plot illustrating the distribution of DEGs, highlighting significantly upregulated (red) and downregulated genes (blue).
