# Supplementary material for: TNBC Spatial Transcriptomic Analysis across Clinical States Reveals Subtype-Specific Networks and Immunosuppressive Niches
Source: Cancer Res Commun. 2026 May 29;6(5):1246–60. doi: 10.1158/2767-9764.CRC-25-0808 (PMC13245550; doi:10.1158/2767-9764.CRC-25-0808)
Supplement: Supplementary Figure 2 — Transcriptional Heterogeneity of all nonmetastatic and metastatic primary tumors. [file crc-25-0808_supplementary_figure_2_suppsf2.docx]

**Supplementary Figure 2.** Transcriptional Heterogeneity of all nonmetastatic and metastatic primary tumors. **A**: PCA plot showing the distribution of all tumor and stroma samples. **B**: Heatmap showing the top DEGs between LN metastases and both primary and nonmetastatic primary tumors. **C**: Volcano plot illustrating the distribution of DEGs of LN Metastases, highlighting significantly upregulated (red) and downregulated genes (blue).
