# Supplementary material for: TNBC Spatial Transcriptomic Analysis across Clinical States Reveals Subtype-Specific Networks and Immunosuppressive Niches
Source: Cancer Res Commun. 2026 May 29;6(5):1246–60. doi: 10.1158/2767-9764.CRC-25-0808 (PMC13245550; doi:10.1158/2767-9764.CRC-25-0808)
Supplement: Supplementary Figure 4 — Diverse immune cell proportions between primary tumors and LN metastases. [file crc-25-0808_supplementary_figure_4_suppsf4.docx]

**Supplementary Figure 4**. Diverse immune cell proportions between primary tumors and LN metastases. **A**: Stacked bar plot of mixed cell populations estimated by SpatialDecon. **B**: Stacked bar plot depicting infiltrating immune cells inferred by CYBERSORTx. **C**: Boxplot showing the differences of immune checkpoint genes expression among primary non-metastatic tumors, primary metastatic tumors, and LN metastases.
