# Supplementary material for: TNBC Spatial Transcriptomic Analysis across Clinical States Reveals Subtype-Specific Networks and Immunosuppressive Niches
Source: Cancer Res Commun. 2026 May 29;6(5):1246–60. doi: 10.1158/2767-9764.CRC-25-0808 (PMC13245550; doi:10.1158/2767-9764.CRC-25-0808)
Supplement: Supplementary Figure 5 — Molecular heterogeneity across TNBC subtypes in two sites. [file crc-25-0808_supplementary_figure_5_suppsf5.docx]

**Supplementary Figure 5**. Molecular heterogeneity across TNBC subtypes in two sites. **A**: PCA plot showing the distribution of all TNBC subtypes in primary and lymph node metastasis. **B**: Kaplan–Meier curves comparing overall survival across TNBC subtypes. P value obtained using the permutation version of the log-rank test.
