# Supplementary material for: TNBC Spatial Transcriptomic Analysis across Clinical States Reveals Subtype-Specific Networks and Immunosuppressive Niches
Source: Cancer Res Commun. 2026 May 29;6(5):1246–60. doi: 10.1158/2767-9764.CRC-25-0808 (PMC13245550; doi:10.1158/2767-9764.CRC-25-0808)
Supplement: Supplementary Figure 6 — Spatial transcriptomics reveals divergence in immune phenotype and functional programs within the same TNBC subtypes. [file crc-25-0808_supplementary_figure_6_suppsf6.docx]

**Supplementary Figure 6**. Spatial transcriptomics reveals divergence in immune phenotype and functional programs within the same TNBC subtypes. **A**: box plots showing differences in infiltrating immune cells among spatial subgroups. **B**-**D**: box plots showing differences in gene expression signatures among combined TNBC subtypes and spatial subgroups. **E**: Bubble plot showing differences in gene expression signatures among ISMS subgroups from Wang et, al. study. **F**: box plots showing differences in immune checkpoint genes across three spatial phenotypes. **G**: box plots showing differences in immune checkpoint genes among TNBC subtypes. **H**: box plots showing differences in immune checkpoint genes among cells among combined TNBC subtypes and spatial subgroups. **I**: Heatmap showing the top DEGs between metastatic primary and nonmetastatic primary TNBC tumors (Excluding HER2+). **J**: Enriched Pathway of DEGs between metastatic primary and nonmetastatic primary TNBC tumors (Excluding HER2+). **K**: Heatmap showing the correlations of expressional signatures in metastatic primary TNBC tumors (Excluding HER2+). **L**: Expression of ERBB2/ HER2 in HER2+ and TNBC samples.  *P* value obtained using the Wilcoxon rank sum test. **M**: Kaplan–Meier curves comparing overall survival across tumors in three clinical stages. Statistical differences across subtypes were assessed using Anova tests and Wilcoxon rank sum test (when comparing each subtype to each of the others. *p < 0.05 and ≥0.01, **p < 0.01 and ≥0.001, ***p<0.001 and ≥0.0001)
