# Supplementary material for: TNBC Spatial Transcriptomic Analysis across Clinical States Reveals Subtype-Specific Networks and Immunosuppressive Niches
Source: Cancer Res Commun. 2026 May 29;6(5):1246–60. doi: 10.1158/2767-9764.CRC-25-0808 (PMC13245550; doi:10.1158/2767-9764.CRC-25-0808)
Supplement: Supplementary Figure 3 — Molecular heterogeneity across TNBC subtypes. [file crc-25-0808_supplementary_figure_3_suppsf3.docx]

**Supplementary Figure 3.** Molecular heterogeneity across TNBC subtypes. A Heatmap showing the correlations of TNBC subtypes for each sample.
